# Distribution of Per- and Polyfluoroalkyl Substances (PFASs) in a Waste-to-Energy Plant—Tracking PFASs in Internal Residual Streams

**DOI:** 10.1021/acs.est.3c10221

**Published:** 2024-04-30

**Authors:** Sofie Björklund, Eva Weidemann, Stina Jansson

**Affiliations:** † Department of Chemistry, Umeå University, SE-901 87 Umeå, Sweden; ‡ Industrial Doctoral School, Umeå University, SE-901 87 Umeå, Sweden

**Keywords:** PFASs, municipal solid waste incineration, fly ash, flue gas treatment, condensate

## Abstract

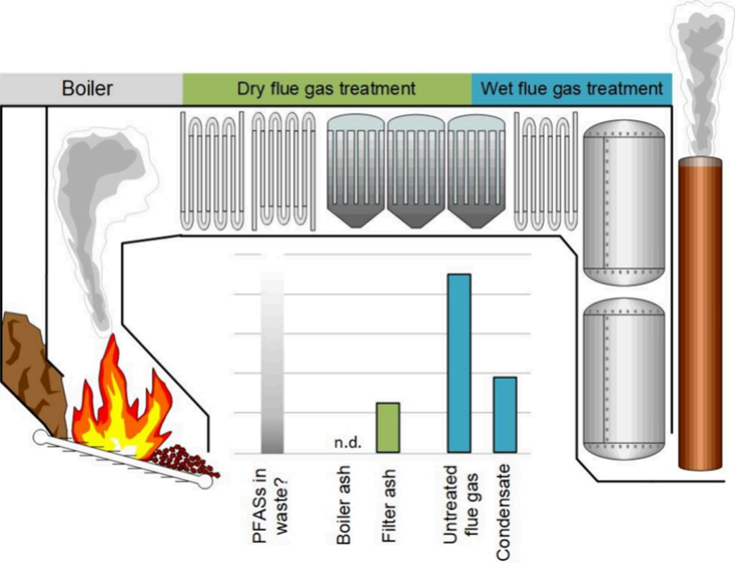

Per- and polyfluoroalkyl substances (PFASs) constitute a diverse group of man-made chemicals characterized by their water- and oil-repellent properties and persistency. Given their widespread use in consumer products, PFASs will inevitably be present in waste streams sent to Waste-to-Energy (WtE) plants. We have previously observed a subset of PFASs in residual streams (ashes, treated process water, and flue gas) from a WtE plant. However, the transport and distribution of PFASs inside the WtE plant have remained unaddressed. This study is part of a comprehensive investigation to create a synoptic overview of the distribution of PFASs in WtE residues. PFASs were found in all sample types except for boiler ash. The total levels of 18 individual PFASs (Σ_18_PFASs) in untreated flue gas ranged from 5.2 to 9.5 ng m^–3^, decreasing with 35% ± 10% after wet flue gas treatment. Σ_18_PFASs in the condensate ranged from 46 to 50 ng L^–1^, of which perfluorohexanoic acid (PFHxA) made up 90% on a ng L^–1^ basis. PFHxA was also dominant in filter ash, where Σ_18_PFASs ranged from 0.28 to 0.79 ng g^–1^. This study shows that flue gas treatment can capture some PFASs and transfer them into WtE residues.

## Abstract 2

Limited research exists on the fate of per- and polyfluoroalkyl substances (PFASs) in Waste-to-Energy (WtE) processes. This study demonstrates that flue gas treatment can capture some PFASs and transfer them to WtE residues.

## Introduction

Per- and polyfluoroalkyl substances (PFASs) constitute a ubiquitous group of man-made chemicals utilized in manufacturing processes and in various consumer products, such as surface treatments for textiles, nonstick products, and food contact materials.^1,2^ The definition of PFASs has varied over time; however, the most recent definition by the OECD from 2021 includes any compound (with a few exceptions) that at least contains a −CF_3_ or a −CF_2_– group.^3^ Due to the strength of the C–F bonds, PFASs are resistant to physical and chemical degradation.^1^ This characteristic renders them suitable for numerous commercial and industrial applications and also means that they are long-lived in the environment, either in their own capacity^4,5^ or as precursors to other, more persistent PFASs.^6−9^ The persistency of PFASs, in combination with their omnipresence in the environment, makes them substances of pressing environmental concern.^10,11^

Given their prevalent use in consumer products, PFASs find their way into waste streams. While landfills have been extensively studied as recognized sources of PFAS contamination in the environment,^12−16^ full-scale waste incineration remains a relatively underexplored area.^16−18^ Recent research has shown the presence of PFASs in fly ash and bottom ash from municipal solid waste incineration (MSWI), in China and USA,^15,19^ and in air near to a MSWI plant in China.^20^ However, these studies have focused on only a few of the residual streams from waste incineration, and comprehensive studies are still scarce. In fact, our literature search only generated two peer-reviewed publications addressing the fate of PFASs in full-scale incineration. One of these investigated the fate of PFASs in a sewage sludge incinerator at a wastewater treatment plant and found a thermal removal efficiency of 51%, indicating that PFASs were incompletely degraded.^21^ The second study was published recently by our research group and investigated the emission of PFASs via residues from MSWI with energy recovery—Waste-to-Energy (WtE).^22^ PFASs were found in bottom ash and treated process water, as well as in flue gas.^22^ That study marked the first report on PFASs in flue gases from full-scale WtE and estimated the annual release of PFASs from the plant at 13 g. However, that number increased to 47 g when 5–8 wt % of sewage sludge was included in the fuel mix.

While our previous study focused on the importance of WtE residues as a secondary release route of PFASs, the primary purpose of this study was to understand the transport and distribution of PFASs inside the WtE plant. The specific objectives of this study were to create a synoptic overview—a “snapshot”—of how PFASs distribute between internal residues and to evaluate the individual contribution of each PFAS to the external residual streams. Additionally, we assessed the efficacy of the wet flue gas treatment in capturing PFASs.

## Materials and Methods

Analytical standards of 9 isotope-labeled PFASs and 18 native PFASs, including C4–C14 perfluorocarboxylic acids (PFCAs), C4–C12 perfluorosulfonic acids (PFSAs), fluorotelomer sulfonic acids (FTSAs), and polyfluoroalkyl phosphoric acid diesters (diPAPs) (see full list in Table S3) were obtained from Wellington Laboratories (Guelph, ON, Canada). Ultrapure water was prepared using a Milli-Q Advantage system (Millipore, Billerica, USA). Glacial acetic acid (100%) was obtained from Merck (Darmstad, Germany), ammonium acetate from Fluka (Buchs, Switzerland), and ammonium hydroxide and hydrochloric acid from J.T. Baker (Phillipsburg, NJ, United States). Methanol was purchased from Fisher Scientific (Leicestershire, UK) and sodium hydroxide from VWR International (Radnor, PA, USA). Activated carbon discs (47 mm × 2 mm) were supplied by Futamura Chemical Co., Ltd. (Nagoya, Aichi, Japan), GF/A filters (47 mm diameter, 1.6 μm pore size) by VWR International (Radnor, PA, USA), and Sartolon polyamide filters (47 mm diameter, pore size 0.45 μm) from Sartorius (Göttingen, Germany). Polyurethane foam filters (47 mm diameter, 50 mm height) were obtained from Sunde Skumplast AS (Gan, Norway). Weak anion exchange solid-phase extraction (WAX-SPE) cartridges (Oasis WAX, 6 cc, 150 mg sorbent, 30 μm particle size) were purchased from Waters Corp. (Milford, MA, USA).

### Study Design

Sampling was carried out at a full-scale WtE plant in northern Sweden. The plant uses a moving grate boiler to incinerate an average of 20 tonnes of waste per hour. The waste fuel consists of 60% residual waste (i.e., waste after sorting out recyclables and food waste) from households and 40% industrial waste and is incinerated at a minimum temperature of 850 °C for 2 s. The plant strictly adheres to legislative demands regarding incineration temperature, residence time, and emission limits. Further details are provided in Section S1 in the SI.

The sampling was performed during two campaigns, consisting of 3 days each. During the first campaign, the waste fuel was incinerated as received (campaign hereafter referred to as municipal solid waste incineration, MSWI). During the second campaign, wastewater treatment sludge (15–20 drywt %) was mixed with the waste fuel at ca. 5–8% on wet basis (hereafter referred to as Sludge:MSWI).

**Figure 1:**
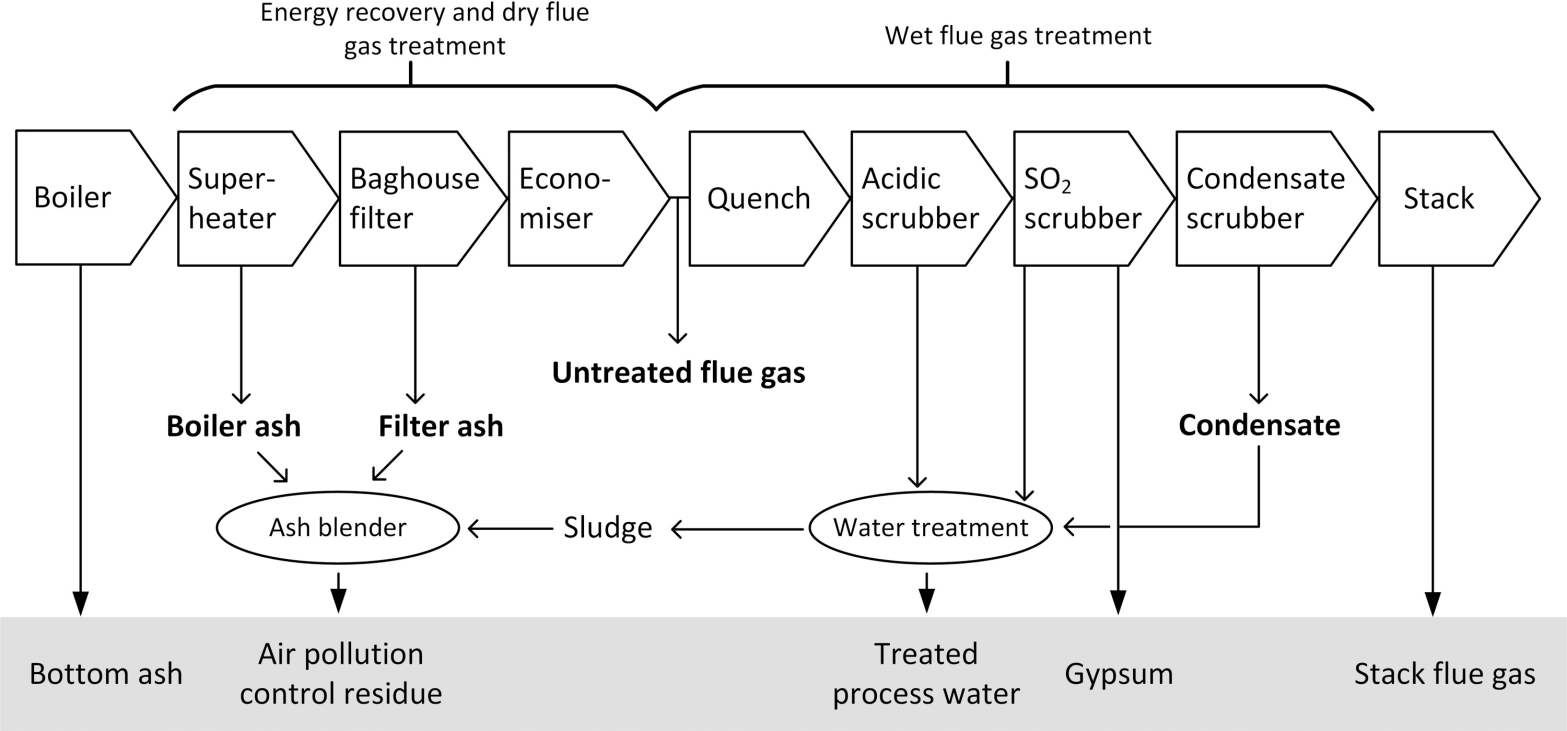
Overview of the WtE process. Text in bold denotes samples collected. Text in gray box represents external residual streams included in the study of Björklund et al.^22^

Condensate, boiler ash, and filter ash were collected and pooled on three occasions per sampling day (Table S2). Flue gas was sampled before the wet flue gas cleaning (Figure 1) using the EN 1948:1 cooled probe sampling train (Figure S1), which was modified with additional features from air-sampling methodologies described elsewhere.^22^ Sludge from the internal water treatment was not accessible at the time of sample collection.

### Sample Preparation

Sample preparation has been described in detail elsewhere.^22,23^ Briefly, boiler ash and filter ash were weighed into a polypropylene tube and spiked with 3 ng internal standard. To each tube, 5 mL of methanol was added before vortexing for 10 s. Thereafter, samples were ultrasonicated for 15 min before being centrifuged for 10 min at 1200*g*. The supernatant was transferred to another tube, and extraction was repeated once more. Extracts were combined, and the volume was reduced to 1 mL under a gentle stream of nitrogen and thereafter diluted to 10 mL with ultrapure Milli-Q water. pH was adjusted to 4 using acetic acid before solid-phase extraction using the protocol described below.

The condensate was spiked with 3 ng internal standard, and pH was adjusted to 4 using acetic acid before solid-phase extraction.

For the flue gas samples, the Milli-Q phase, sodium hydroxide phase, and filters (i.e., activated carbon discs and polyurethane foam) were each spiked with 3 ng internal standard. The filters were extracted using the same protocol as for the ash but using 50 mL of methanol. The Milli-Q phase was adjusted to pH 4 using acetic acid and filtered using GF/A filters to remove precipitate. The sodium hydroxide phase was adjusted to pH 4 using hydrochloric acid and thereafter filtered using first GF/A filters and followed by polyamide filters to remove the precipitate. The GF/A filters were extracted separately using 10 mL of methanol which was combined with the respective sample before extraction.

Samples were extracted using WAX-SPE according to the ISO25101 methodology, with minor modifications.^21,23^ First, cartridges were conditioned using 4 mL each of 0.1% ammonium hydroxide in methanol, methanol, and Milli-Q water. Thereafter, samples were loaded at a rate of approximately one drop per second, and the cartridges were rinsed using 4 mL of 25 mM ammonium acetate buffer and Milli-Q each. The cartridges were dried under vacuum for 30 min before elution in two fractions of 4 mL methanol and 4 mL 0.1% ammonium hydroxide in methanol (only the second fraction is presented herein).

The extracts were reduced to 150 μL under a gentle nitrogen flow and transferred to a LC vial using methanol to a final volume of 500 μL. After the addition of 3 ng recovery standard, 80 μL of extract was combined with 120 μL of 2 mM ammonium acetate in a vial for analysis.

### Analysis

Analysis was performed using a 6560 Ion Mobility Q-ToF LC–MS instrument (Agilent Technologies, USA) with electrospray ionization in negative mode. Data were acquired in targeted MS/MS mode. Separation was achieved on a C18 column (3 μm, 110 Å, 150 × 2.0 mm, Phenomenex, Torrance, CA, USA) using a flow rate of 0.5 mL/min and water–methanol gradient, both containing 2 mM ammonium acetate (additional details in the SI).

### Quality Control

For the flue gas sampling, an isotope-labeled standard was added to the Milli-Q phase prior to sampling, to control for losses during sampling. Polyurethane foam filters and activated carbon discs were washed in methanol for at least 12 h and wrapped in aluminum foil before and after sampling. Impinge bottles and filter holders were precleaned by baking at 550 °C for 5 h and thoroughly rinsed with methanol before sampling. Upon arrival at the lab, the Milli-Q phase and sodium hydroxide phase were transferred to HDPE bottles and stored at −18 °C.

The condensate samples were collected in pre-rinsed high-density polyethylene (HDPE) bottles and stored at −18 °C upon arrival at the lab. Boiler ash and filter ash were collected in stainless steel containers.

Field blanks for untreated flue gas and condensate were collected during each sampling campaign. Low levels of PFASs were detected in the untreated flue gas field blank, while all PFASs were below the limit of detection (LOD) for the condensate (Table S6).

Two procedural blanks consisting of Milli-Q water were included in every batch of samples, making up a total of 18 procedural blanks. In general, no PFASs were detected in the procedural blanks, with a few exceptions (Table S10). The LOD was calculated as three times the procedural blank concentration plus standard deviation (Table S4). If no PFASs were detected, the LOD was calculated as the average instrument noise level plus 3 times the standard deviation. Limit of quantification (LOQ) was calculated as the average instrument noise level plus 10 times the standard deviation.

Quantification was performed using isotope-labeled standards. For compounds where a corresponding isotope-labeled standard was not available, the standard closest in retention time was used for quantification.

The condensate, boiler ash, and filter ash samples were analyzed in triplicate. The flue gas samples did not allow for replicate analysis as the whole volume was consumed during sample preparation. To allow for comparison between samples, the volume of sampled flue gas was normalized to dry gas, 0 °C, and a pressure of 1 atm (additional details in Section S2 in SI).

## Results and Discussion

PFASs were detected in all samples of untreated gas, condensate, and filter ash but not in boiler ash. Short-chain PFCAs were dominant in all sample types, and the most prevalent species, perfluorohexanoic acid (PFHxA), was present in 80% of all samples, followed by perfluorooctanoic acid (PFOA) in 60% of all samples and perfluoroheptanoic acid (PFHpA) in 50% of all samples. To facilitate interpretation, the residual streams were grouped based on where in the process they are generated: (i) energy recovery and dry flue gas treatment or (ii) the wet flue gas treatment.

### Energy Recovery and Dry Flue Gas Treatment

Energy recovery aims to harness the energy produced during incineration to provide district heating and electricity, and dry flue gas treatment serves to remove ash from flue gas. Starting at the superheaters, the boiler ash—the heavier and hotter ash fraction—falls out (Figure 1). The process continues with baghouse filters, where fabric filters capture the fine particles of the filter ash.

No individual PFASs were detected in the boiler ash. The temperature at the boiler outlet where the boiler ash falls out ranges from 700 to 650 °C, which is within the range where defluorination of both PFSAs and PFCAs could occur.^17^ At the baghouse filter where the filter ash is collected (Figure 1), the temperature is considerably lower, around 200 °C, meaning degradation could still occur but at a much smaller extent in comparison to the boiler outlet.^17^ The total concentration of detected PFASs (Σ_18_PFASs) in the filter ash ranged from 0.28 to 0.79 ng g^–1^. PFHxA was the dominant PFAS species in the filter ash (0.28–0.60 ng g^–1^). PFOA was the only other compound detected and was found on 1 out of 3 days, although below LOQ (Table S5). Notably, activated carbon is injected before the fabric filters to capture other incineration-related contaminants. Since activated carbon is used in the remediation of PFAS-contaminated water and soil (see e.g.,^25,26^), it was reasonable to expect that the addition of activated carbon would affect the PFAS content of filter ash. There was also the possibility that the fabric filters, which according to the supplier contain Teflon, could be a contributing factor to the PFASs found in the filter ash.

A small number of studies have investigated the PFAS content of WtE ashes. One study reported finding PFASs in fly ash from three different WtE plants.^19^ Although the identified PFASs and their concentrations varied widely between plants and sampling days, the total levels were considerably higher than those reported in the present study (1.5–88 ng g^–1^ in Liu et al.^19^ vs 0.28–0.79 ng g^–1^ in the present study). PFSAs (mainly perfluorooctanesulfonic acid, PFOS) were reported as the dominant class of PFASs by Liu et al. (2021), while in our study, no PFSAs were found in the filter ash. It is presently unknown whether this is the result of differences in the PFAS content in the waste fuel, different incineration conditions, or both. Studies have reported PFASs in landfill leachate from ash monofills, but since fly ash and bottom ash were combined in these deposits, it is not possible to determine from which ash fraction the detected PFASs were derived.^15,27^

### Wet Flue Gas Treatment

Wet flue gas treatment starts with the quench, the purpose of which is to cool down the flue gas. Untreated flue gas was sampled before the quench (Figure 1) to enable an assessment of the efficiency of the wet flue gas treatment. The quench is followed by an acid scrubber, where water is used to wash out HCl, NH_3_, and Hg from the flue gas, and a SO_2_ scrubber, where a slaked lime mixture (Ca(OH)_2_) is added to capture SO_2_ by forming gypsum (CaSO_4_). Lastly the flue gas passes through a condensate scrubber, where the condensate was collected. Here, some of the excess water and the remaining heat are captured.

**Figure 2:**
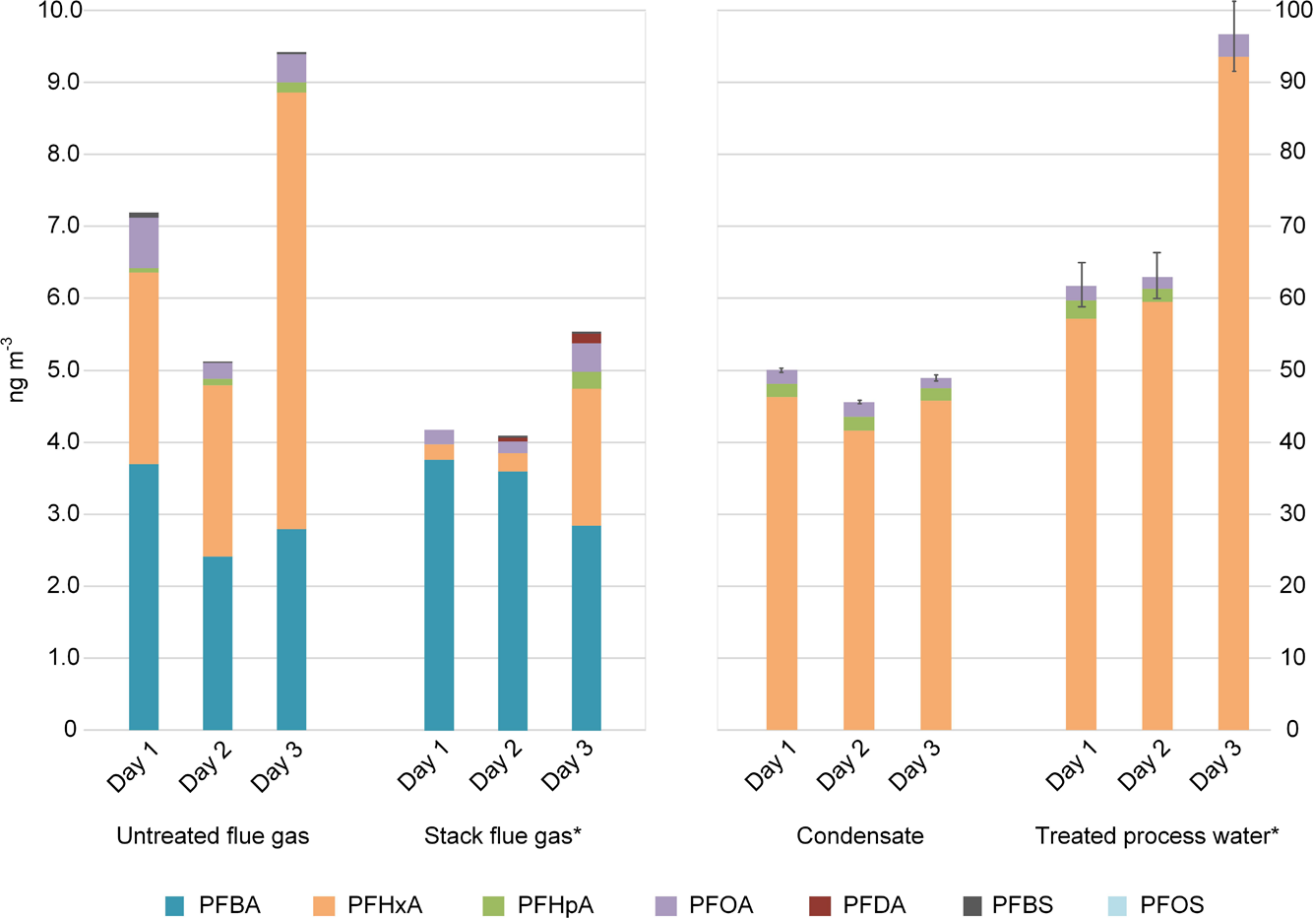
Levels of detected PFASs in untreated flue gas, stack flue gas, condensate, and treated process water during incineration of MSW. Error bars indicate standard deviation of duplicate (condensate) or triplicate (treated process water) samples; see Table S9 for further details. * denotes samples from Björklund et al.^22^

∑_18_PFASs in untreated flue gas ranged from 5.2 to 9.5 ng m^–3^ (Figure 2). Of the detected PFASs, perfluorobutanoic acid (PFBA) and PFHxA were present in the highest concentrations, in ranges of 2.4–3.7 and 2.4–6.1 ng m^–3^, respectively (Table S5). PFHpA, PFOA, and perfluorobutanesulfonic acid (PFBS) were detected in both untreated flue gas and the field blank, in concentrations close to or below LOQ (Tables S4 and S5).

**Table 1:**
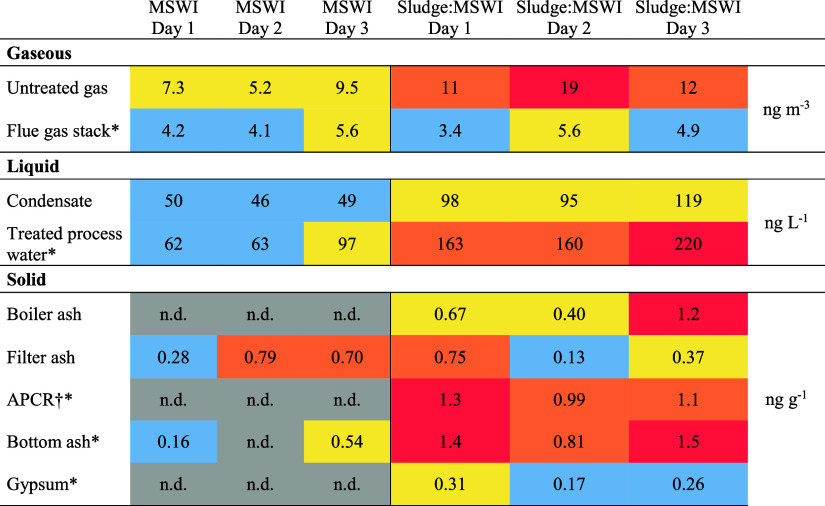
Sum of Detected PFASs^a^

a * denotes samples from Björklund et al. (2023).^22^^†^APCR = air pollution control residue. n.d. = not detected. Gaseous: blue, LOD—5 ng m^–3^, yellow—5.0–10 ng m^–3^, orange—10–15 ng m^–3^, red >15 ng m^–3^. Liquid: blue, LOD—70 ng L^–1^, yellow—70–120 ng L^–1^, orange—120–170 ng L^–1^, red >170 ng L^–1^. Solid: gray < LOD, blue, LOD—0.30 ng g^–1^, yellow—0.30–0.70 ng g^–1^, orange—0.7–1.1 ng g^–1^, red >1.1 ng g^–1^.

Although only one previous study has reported on the presence of PFASs in flue gas from full-scale WtE,^22^ a recent work by Seay et al. (2023) investigated the fate of PFASs in a sewage sludge incinerator.^21^ PFASs were sampled in the stack flue gas, and out of the 30 analyzed PFASs, 30 were detected. Moreover, Seay et al. (2023) suggest that, although some PFASs (e.g., PFHxA and PFOS) had negative net mass flows (i.e., the PFAS concentration was lower in effluent streams than influent ones), the PFASs were overall incompletely degraded. PFASs have also been detected in the ambient air at two incineration plants in China,^20^ where both neutral and ionic PFASs were detected in both locations. Of the ionic PFASs, PFBA was dominant in one location and PFOA in the other. The study did not discuss whether the source of the PFASs was the unincinerated waste or the incineration process itself.

In the condensate, which is generated at the final stage of the wet flue gas treatment, ∑_18_PFASs ranged from 46 to 50 ng L^–1^. Consistent with the untreated flue gas, PFHxA was the most prevalent, constituting approximately 90% on a ng L^–1^ basis (Figure 2). Additionally, PFHpA, PFOA, and perfluorodecanoic acid (PFDA) were detected, but at significantly lower concentrations, falling within the low ng L^–1^ range (Table S5). Notably, the levels of PFASs in the leachate from the waste stockpile at the same WtE plant were 4 to 5 times higher (172–262 ng L^–1^) than the condensate, of which short-chain PFCAs made up 60–70% of the concentration in all samples.^24^ However, the identified PFASs in the leachate from the waste stockpile only made up 12 ± 4% of the extractable organofluorine (EOF), which indicates that there are other PFASs in the leachate that could not be quantified by targeted analysis.^24^ Since the unidentified PFASs could be assumed to originate from the waste, these will also be subjected to thermal degradation during incineration and could form degradation products that remain to be identified.

Surface water collected at the outlet of a Swedish hazardous waste management facility was found to contain PFASs in concentrations more than 30 times higher (1700 ng L^–1^) than the condensate, of which PFSAs were predominant.^28^ It was not specified whether waste incineration takes place at this facility.

### Internal versus External Residual Streams

This study is part of a comprehensive investigation into the fate of PFASs in a WtE plant. In a previous publication, the external residual streams from the same WtE plant, as was investigated in this study, were assessed.^22^ This study adds to the findings by providing insights regarding the transport and distribution of PFASs internally in the WtE plant.

Air pollution control residue (APCR) is an external residue stream composed of a mixture of filter ash, boiler ash, and sludge from a WtE plant’s internal water treatment (Figure 1). No PFASs were found in APCR and boiler ash (Table 1). Conversely, PFHxA and PFOA were detected in the filter ash (Table S5). One plausible reason for finding PFHxA and PFOA in the filter ash but not the APCR is the dilution effect of the water treatment sludge (Figure 1).

∑_18_PFASs in the treated process water (i.e., effluent water after the *in-house* water treatment; description of the water treatment can be found in the SI) were found to be 25–100% higher in comparison to the condensate (Figure 2). This suggests that there are additional sources of PFASs introduced to the water treatment that contribute to the level of ∑_18_PFASs in the treated process water. The observed increase in ∑_18_PFASs could originate from the acidic scrubber (Figure 1) or be a result of transformation of precursor PFASs during the water treatment. To fully understand the occurrence and distribution of PFASs in the water treatment facility and identify potential additional routes for PFASs into treated process water, a more thorough investigation of the water treatment process is required.

In the stack flue gas, the concentration of ∑_18_PFASs ranged from 4.1 to 5.6 ng m^–3^; for the untreated gas, this range was 5.2–9.5 ng m^–3^. The reduction of ∑_18_PFASs in the stack flue gas was, on average, −35% ± 10% on a ng m^–3^ basis. While PFBA did not exhibit a distinct decrease (+16% ± 22% on a ng m^–3^ basis), the average reduction of PFHxA was −83% ± 10% after the wet flue gas treatment (Figure 2).

Given the low temperatures (below 100 °C) in the wet flue gas treatment, it is improbable that degradation plays a substantial role in the observed reduction of PFASs following wet flue gas treatment. Instead, it is more likely that PFASs are removed during various stages of the wet flue gas treatment and directed to other internal streams. The notable decrease in PFHxA in the stack flue gas, coupled with its predominance in the condensate (Figure 2), strongly suggests that PFHxA is transferred from the flue gas into the condensate in the condensate scrubber. However, the absence of PFBA in the condensate, along with its insignificant decline in the stack flue gas, indicates that the condensation scrubber may not be an efficient process step for the removal of all PFASs.

### Sludge:MSWI versus MSWI

The sampling was carried out in two campaigns; during one, the plant was incinerating MSW as received, and during the other MSW was being coincinerated with the sewage sludge from the local wastewater treatment plant. Sludge coincineration is not a standard operation at this particular plant, but this provided us with the opportunity to carry out sampling during incineration of a fuel type that is well known to contain PFASs.^9,29^

With the exception of stack flue gas and filter ash, ∑_18_PFASs exhibited higher levels for Sludge:MSWI than MSWI in all matrices (Table 1). Specifically, the average concentration of ∑_18_PFASs in untreated gas was twice as high during Sludge:MSWI as compared to MSWI (Table 1). In both cases, PFBA and PFHxA were the predominant PFASs detected. The average concentration of PFBA remained consistent across both campaigns, measuring on average 2.9 ng m^–3^ during Sludge:MSWI and 3.0 ng m^–3^ during MSWI. In contrast, the average concentration of PFHxA was 2–3 times higher during Sludge:MSWI in comparison to MSWI, measuring on average 9.3 and 3.7 ng m^–3^, respectively. Although this increased abundance of PFHxA during Sludge:MSWI is likely attributable to the presence of PFHxA or its precursors in the fuel, it is not possible to definitively determine whether it originated from the sludge or the MSW.

The average ∑_18_PFASs in the condensate was 48 ng L^–1^ during MSWI and 110 ng L^–1^ during Sludge:MSWI. Similar to the untreated flue gas, the concentration of PFHxA doubled during the coincineration of sludge (Table 1).

In filter ash, the average ∑_18_PFASs was slightly higher during MSWI compared to Sludge:MSWI (0.59 vs 0.43 ng g^–1^, respectively), while for all other matrices ∑_18_PFASs were higher during Sludge:MSWI. PFHxA was detected in all filter ash samples during both MSWI and Sludge:MSWI, at 0.28–0.70 and 0.13–0.40 ng g^–1^, respectively. Additionally, PFOA was detected once during the MSWI campaign (0.18 ng g^–1^) and once during Sludge:MSWI (0.38 ng g^–1^); however, both values were below LOQ. No PFASs were detected in the boiler ash during MSWI. During Sludge:MSWI, PFBA was detected in two of the three samples of boiler ash, but below LOQ (0.67 and 0.40 ng g^–1^, respectively), and PFBS was detected in one of the three samples (1.2 ng g^–1^).

Combined, these findings highlight the impact of fuel composition on the presence of PFASs in waste incineration residues. While coincineration of sewage sludge may not be a standard practice across most WtE facilities, it is of critical importance to be aware of the contribution of additional, potentially PFAS-contaminated, waste types to the prevalence of PFASs.

### Implications for Waste-to-Energy and Future Perspectives

There is a critical need for safe disposal of products containing PFASs.^16−18^ Despite their continuous presence in consumer products since the 1950s,^30^ our understanding of how PFASs behave in full-scale waste incineration processes is limited.^18^ Until recently, research efforts have primarily focused on lab-scale assessments of the degradation of individual materials or compounds, under highly controlled and simplified conditions.^16,17,31^ While lab-scale studies are crucial for understanding the thermal degradation mechanisms of PFASs, it remains imperative to investigate real-world operations in full-scale facilities.

In the present study, short-chain PFCAs were primarily detected along with PFOA, PFDA, and PFBS, which were often below or close to LOQ. The presence of short-chain PFCAs in the WtE residues could be the result of (i) presence of precursors in the waste that degraded to the detected compound or (ii) presence of the parent compound itself in the waste. Several studies have investigated the degradation of PFASs under various conditions, and results sometimes diverge significantly. For example, one study found increasing thermal stability of PFCAs with increasing chain length,^32^ while another study reported short-chain PFCAs as the degradation product of longer PFCAs.^33^ The same studies also reported conflicting results on the relative stability of PFCAs and PFSAs, with Watanabe et. al (2016) reporting PFCAs to be more stable at a given temperature in relation to PFSAs,^33^ while Xiao et. al (2020) found the opposite relationship.^32^ There is also a lack of agreement between several computational and lab-scale studies,^31^ which further highlights the complexity of combustion chemistry and the need to combine computational, lab-scale, and full-scale studies to advance the understanding of the fate of PFASs in WtE processes.

This study, in conjunction with previously published research,^22^ provides a synoptic overview of the distribution of PFASs in both external and internal residual streams. These studies also emphasize the significance of fuel composition for the occurrence of PFASs in waste incineration residues. Additionally, they contribute with initial insights into the impact of flue gas treatment on the capture of PFASs. Notably, despite the ∑_18_PFAS concentration in untreated flue gas doubling during the coincineration of sludge, the concentration in stack flue gas remained the same (Table 1). The ability of the wet flue gas treatment to capture PFASs seems to differ between compounds (e.g., PFHxA decreased while PFBA did not). However, too few compounds were detected to conclude which characteristics could be favorable for capture by the flue gas treatment. Further research is required to establish how the properties of individual PFASs influence their removal from flue gas into residual streams.

Future studies should endeavor to bridge the gap between full-scale and lab-scale investigations in order to gain a deeper understanding of how PFASs behave in WtE processes. This could involve expanding the list of analytes to encompass additional degradation products and volatile species, as well as establishing a mass-balance under conditions that are relevant for WtE. Importantly, volatile organofluorine has been reported repeatedly as thermal degradation products of PFASs,^17,31^ and future studies should endeavor to map the potential release of such compounds from full-scale WtE. Lastly, more efforts are needed to make flue gas sampling and analysis of PFASs commercially accessible for waste incineration operators.

## Supporting Information Available

Detailed description of the WtE plant, flue gas sampling procedure, analytical conditions, and quality control as well as concentration of PFASs in individual samples (PDF)

## References

[ref1] Kissa E. Fluorinated Surfactants and Repellents, 2nd ed.; Marcel Dekker: New York, NY, 2001.

[ref2] Glüge J.; Scheringer M.; Cousins I. T.; DeWitt J. C.; Goldenman G.; Herzke D.; Lohmann R.; Ng C. A.; Trier X.; Wang Z. An overview of the uses of per- and polyfluoroalkyl substances (PFAS). Environ. Sci.: Processes Impacts 2020, 22 (12), 2345–2373. 10.1039/D0EM00291G. PMC7784712 33125022

[ref3] OECD. Reconciling Terminology of the Universe of Per- and Polyfluoroalkyl Substances: Recommendations and Practical Guidance - Series on Risk Management, No. 61, 2021; Vol. ENV/CBC/MONO(2021)25.

[ref4] OECD Co-operation on Existing Chemicals - Hazard Assessment of Perfluorooctane Sulfonate and its Salts. Environment Directorate Joint Meeting of the Chemicals Committe and the Working Party on Chemicals, Pesticides and Biothechnology, Organisation for Economic Co-operation and Development, 2002; Vol. ENV/JM/RD(2002)17/FINAL.

[ref5] Ruyle B. J.; Thackray C. P.; Butt C. M.; LeBlanc D. R.; Tokranov A. K.; Vecitis C. D.; Sunderland E. M. Centurial Persistence of Forever Chemicals at Military Fire Training Sites. Environ. Sci. Technol. 2023, 57 (21), 8096–8106. 10.1021/acs.est.3c00675. 37184088 PMC10233753

[ref6] Houtz E. F.; Sedlak D. L. Oxidative conversion as a means of detecting precursors to perfluoroalkyl acids in urban runoff. Environ. Sci. Technol. 2012, 46 (17), 9342 10.1021/es302274g. 22900587

[ref7] Houtz E. F.; Higgins C. P.; Field J. A.; Sedlak D. L. Persistence of Perfluoroalkyl Acid Precursors in AFFF-Impacted Groundwater and Soil. Environ. Sci. Technol. 2013, 47 (15), 8187–8195. 10.1021/es4018877. 23886337

[ref8] Washington J. W.; Jenkins T. M.; Rankin K.; Naile J. E. Decades-Scale Degradation of Commercial, Side-Chain, Fluorotelomer-Based Polymers in Soils and Water. Environ. Sci. Technol. 2015, 49 (2), 915–923. 10.1021/es504347u. 25426868

[ref9] Eriksson U.; Haglund P.; Kärrman A. Contribution of precursor compounds to the release of per-and polyfluoroalkyl substances(PFASs) from waste water treatment plants(WWTPs). J. Environ. Sci. 2017, 61 (11), 80–90. 10.1016/j.jes.2017.05.004. 29191318

[ref10] Cousins I. T.; DeWitt J. C.; Glüge J.; Goldenman G.; Herzke D.; Lohmann R.; Ng C. A.; Scheringer M.; Wang Z. The high persistence of PFAS is sufficient for their management as a chemical class. Environ. Sci.: Processes Impacts 2020, 22 (12), 2307–2312. 10.1039/D0EM00355G. PMC7784706 33230514

[ref11] Kurwadkar S.; Dane J.; Kanel S. R.; Nadagouda M. N.; Cawdrey R. W.; Ambade B.; Struckhoff G. C.; Wilkin R. Per- and polyfluoroalkyl substances in water and wastewater: A critical review of their global occurrence and distribution. Sci. Total Environ. 2022, 809, 151003 10.1016/j.scitotenv.2021.151003. 34695467 PMC10184764

[ref12] Chen Y.; Zhang H.; Liu Y.; Bowden J. A.; Tolaymat T. M.; Townsend T. G.; Solo-Gabriele H. M. Evaluation of per- and polyfluoroalkyl substances (PFAS) in leachate, gas condensate, stormwater and groundwater at landfills. Chemosphere 2023, 318, 137903 10.1016/j.chemosphere.2023.137903. 36669537 PMC10536789

[ref13] Knutsen H.; Mæhlum T.; Haarstad K.; Slinde G. A.; Arp H. P. H. Leachate emissions of short- and long-chain per- and polyfluoralkyl substances (PFASs) from various Norwegian landfills. Environ. Sci.: Processes Impacts 2019, 21 (11), 1970–1979. 10.1039/C9EM00170K. 31411188

[ref14] Fuertes I.; Gómez-Lavín S.; Elizalde M. P.; Urtiaga A. Perfluorinated alkyl substances (PFASs) in northern Spain municipal solid waste landfill leachates. Chemosphere 2017, 168 (C), 399–407. 10.1016/j.chemosphere.2016.10.072. 27810540

[ref15] Solo-Gabriele H. M.; Jones A. S.; Lindstrom A. B.; Lang J. R. Waste type, incineration, and aeration are associated with per- and polyfluoroalkyl levels in landfill leachates. Waste manage. 2020, 107, 191–200. 10.1016/j.wasman.2020.03.034. PMC8335518 32304853

[ref16] Stoiber T.; Evans S.; Naidenko O. V. Disposal of products and materials containing per- and polyfluoroalkyl substances (PFAS): A cyclical problem. Chemosphere 2020, 260, 127659 10.1016/j.chemosphere.2020.127659. 32698118

[ref17] Longendyke G. K.; Katel S.; Wang Y. PFAS fate and destruction mechanisms during thermal treatment: a comprehensive review. Environ. Sci.: Processes Impacts 2022, 24 (2), 196–128. 10.1039/D1EM00465D. 34985474

[ref18] Ng C.; Cousins I. T.; DeWitt J. C.; Glüge J.; Goldenman G.; Herzke D.; Lohmann R.; Miller M.; Patton S.; Scheringer M.; Trier X.; Wang Z. Addressing Urgent Questions for PFAS in the 21st Century. Environ. Sci. Technol. 2021, 55 (19), 12755–12765. 10.1021/acs.est.1c03386. 34519210 PMC8590733

[ref19] Liu S.; Zhao S.; Liang Z.; Wang F.; Sun F.; Chen D. Perfluoroalkyl substances (PFASs) in leachate, fly ash, and bottom ash from waste incineration plants: Implications for the environmental release of PFAS. Sci. Total Environ. 2021, 795, 148468 10.1016/j.scitotenv.2021.148468. 34252761

[ref20] Wang B.; Yao Y.; Chen H.; Chang S.; Tian Y.; Sun H. Per- and polyfluoroalkyl substances and the contribution of unknown precursors and short-chain (C2–C3) perfluoroalkyl carboxylic acids at solid waste disposal facilities. Sci. Total Environ. 2020, 705, 135832 10.1016/j.scitotenv.2019.135832. 31831231

[ref21] Seay B. A.; Dasu K.; MacGregor I. C.; Austin M. P.; Krile R. T.; Frank A. J.; Fenton G. A.; Heiss D. R.; Williamson R. J.; Buehler S. Per- and polyfluoroalkyl substances fate and transport at a wastewater treatment plant with a collocated sewage sludge incinerator. Sci. Total Environ. 2023, 874, 162357 10.1016/j.scitotenv.2023.162357. 36858229

[ref22] Björklund S.; Weidemann E.; Jansson S. Emission of Per- and Polyfluoroalkyl Substances from a Waste-to-Energy Plant - Occurrence in Ashes, Treated Process Water, and First Observation in Flue Gas. Environ. Sci. Technol. 2023, 57 (27), 10089–10095. 10.1021/acs.est.2c08960. 37319344 PMC10339719

[ref23] Awad R.; Johann Bolinius D.; Strandberg J.; Yang J.-J.; Sandberg J.; Adeoye Bello M.; Gobelius L.; Egelrud L.; Härnwall E.-L. PFAS in waste residuals from Swedish incineration plants - IVL Report No. B 2422, 2021.

[ref24] Björklund S.; Weidemann E.; Yeung L. W.; Jansson S. Occurrence of per- and polyfluoroalkyl substances and unidentified organofluorine in leachate from waste-to-energy stockpile - A case study. Chemosphere 2021, 278, 130380 10.1016/j.chemosphere.2021.130380. 33823356

[ref25] Cantoni B.; Turolla A.; Wellmitz J.; Ruhl A. S.; Antonelli M. Perfluoroalkyl substances (PFAS) adsorption in drinking water by granular activated carbon: Influence of activated carbon and PFAS characteristics. Sci. Total Environ. 2021, 795, 148821 10.1016/j.scitotenv.2021.148821. 34252781

[ref26] Niarchos G.; Ahrens L.; Kleja D. B.; Leonard G.; Forde J.; Bergman J.; Ribeli E.; Schütz M.; Fagerlund F. n-situ application of colloidal activated carbon for PFAS-contaminated soil and groundwater: A Swedish case study. Remediation 2023, 33 (2), 101–110. 10.1002/rem.21746.

[ref27] Liu Y.; Mendoza-Perilla P.; Clavier K. A.; Tolaymat T. M.; Bowden J. A.; Solo-Gabriele H. M.; Townsend T. G. Municipal solid waste incineration (MSWI) ash co-disposal: Influence on per- and polyfluoroalkyl substances (PFAS) concentration in landfill leachate. Waste manage. 2022, 144, 49–56. 10.1016/j.wasman.2022.03.009. PMC10536760 35306465

[ref28] Björnsdotter M.; Yeung L. W.-Y.; Kärrman A.; Ericson Jogsten I. Ultra-short-chain perfluoroalkyl acids including trifluoromethane sulfonic acid (TFMS) in water connected to known and suspected point sources in Sweden. Environ. Sci. Technol. 2019, 53 (19), 11093–11101. 10.1021/acs.est.9b02211. 31496234

[ref29] Kärrman A.; Wang T.; Kallenborn R.; Langseter A. M.; Gro̷nhovd S. M.; Ræder E. M.; Lyche J. L.; Yeung L.; Chen F.; Eriksson U.; Aro A.; Fredriksson F. PFASs in the Nordic environment: Screening of Poly- and Perfluoroalkyl Substances (PFASs) and Extractable Organic Fluorine (EOF) in the Nordic Environment; TemaNord, 2019; ISSN 0908-6692-2019:515.

[ref30] Lindstrom A. B.; Strynar M. J.; Libelo E. L. Polyfluorinated Compounds: Past, Present, and Future. Environ. Sci. Technol. 2011, 45 (19), 7954–7961. 10.1021/es2011622. 21866930

[ref31] Wang J.; Lin Z.; He X.; Song M.; Westerhoff P.; Doudrick K.; Hanigan D. Critical review of thermal decomposition of per- and polyfluoroalkyl substances: mechanisms and implications for thermal treatment processes. Environ. Sci. Technol. 2022, 56, 5355–5370. 10.1021/acs.est.2c02251. 35446563

[ref32] Xiao F.; Sasi P. C.; Yao B.; Kubátová A.; Golovko S. A.; Golovko M. Y.; Soli D. Thermal stability and decomposition of perfluoroalkyl substances on spent granular activated carbon. Environ. Sci. Technol. Lett. 2020, 7, 343–350. 10.1021/acs.estlett.0c00114.

[ref33] Watanabe N.; Takemine S.; Yamamoto K.; Haga Y.; Takata M. Residual organic fluorinated compounds from thermal treatment of PFOA, PFHxA and PFOS adsorbed onto granular activated carbon (GAC*)*. J. Mater. Cycles Waste Manage. 2016, 18, 625–630. 10.1007/s10163-016-0532-x.

