# Supplementary material for: Distribution of Per- and Polyfluoroalkyl Substances (PFASs) in a Waste-to-Energy Plant—Tracking PFASs in Internal Residual Streams
Source: Environ Sci Technol. 2024 Apr 30;58(19):8457–63. doi: 10.1021/acs.est.3c10221 (PMC11097385; doi:10.1021/acs.est.3c10221)
Supplement: Supplementary file 1 — es3c10221_si_001.pdf [file es3c10221_si_001.pdf]

Supporting Information

Title: Distribution of Per- and Polyfluoroalkyl Substances (PFASs) in a Waste-to-Energy Plant – Tracking PFASs in Internal Residual Streams

Sofie Björklund[a.b], Eva Weidemann[a], Stina Jansson[a *]

[a]Department of Chemistry, Umeå University, SE-901 87 Umeå, Sweden

[b]Industrial Doctoral School for Research and Innovation, Umeå University, SE-901 87 Umeå, Sweden

*Corresponding author

Pages: 15

Figures: 1

Tables: 13

Contents

Section S1. Plant description. 3

Table S1. Emissions of metals, acids, ammonia and dioxins in treated process water and flue gas from the waste incineration plant where sampling was conducted. 4

Section S2. Flue gas sampling. 5

Figure S1. Flue gas sampling train. 6

Equation S1. 7

Section S3. Sampling protocol. 7

Table S2. Sample collection protocol. 7

Section S4. Instrumental parameters, quality assurance 8

Table S3. Target compounds included in the analysis, their chemical formula, parent ion, quantification ion and the corresponding internal and recovery standard. 8

Table S4. Limit of detection per sample matrix. 9

Table S5. Concentrations of PFAS in all samples. 10

Table S6. Concentration of PFAS in field blanks. 11

Table S7. Average internal standard recoveries per sample matrix. 11

Table S8. Recovery of flue gas sampling spikes. 12

Table S9. Relative standard deviation of replicates per sample. 12

Tabell S10. Concentration of PFAS in procedural blanks ($ng \cdot L^{-1}$). 12

Table S11. Flue gas sampling parameters. 14

Table S12. LC-MS instrument parameters. 14

Table S13. LC mobile phase program. 15

References 15

Section S1. Plant description.

The Waste-to-Energy (WtE) plant is utilized for energy recovery from waste, with an uptake area of 150-400 km. The waste fuel (except for a portion of waste that require pre-crushing) is delivered directly into the waste bunker to reduce need for handling from a hygienic and logistic perspective. A schematics of the plant layout can be found in Figure 1 in the main article. A condensed description of the plant operation is given below (a full detailed description can be found in Björklund et. al (2023)[1]).

To distribute moisture content and break up mono-fractions, manual fuel mixing is performed in the waste bunker using the waste handling crane. The crane operator adds approximately four 5-ton loads of fuel to the fuel hopper each hour, from where the moving grate boiler is fed continuously by a hydraulic pusher system. After the incineration (minimum 2 s residence time at 850 °C in accordance to Swedish law, SFS 2013:253 §32) the flue gas goes through two empty passes to reduce temperature (from > 800 °C to ca 600 °C) before reaching the superheaters, where boiler ash is collected. After the superheater, and an economizer, activated carbon is added to the flue gas (220 °C) which is then allowed to pass through textile filters to capture dioxins and ash. After the textile filters, the flue gas passes a second economizer before being quenched with water to reduce the temperature. After the quench, the flue gases pass through an acid scrubber, where water is used to wash out the HCl, NH_3 and Hg from the flue gases. A portion of water from the acid scrubber is directed to the internal water treatment, while the majority is recirculated in the scrubber. The flue gases continue into the SO_2 scrubber, where the flue gases are sprayed with a slaked lime mixture ($Ca(OH)_2$) that forces the SO_2 from the gas phase to form gypsum ($CaSO_4$). After the SO_2 scrubber, the flue gases are led through a condensate scrubber, removing some of the excess water and the remaining heat. Lastly, the flue gases are reheated slightly before they are

released through the stack. All stack gas measuring equipment are placed directly before the gases are released into the air.

During 2021, the plant logged no hours of operations with emissions exceeding legislative limits and had very good adherence to the environmental requirements outlined in Swedish law (SFS 2013:253). In Table S1 the adherence to SFS 2013:253 with regard to emissions of metals, acids, ammonia and dioxins to recipient water and air through treated process water and flue gas is outlined. The emissions are below the legislative limits for all measured contaminants.

Table S1. Emissions of metals, acids, ammonia and dioxins in treated process water and flue gas from the waste incineration plant where sampling was conducted.

	Treated process water (mg·L^{-1})		Flue gas (mg·Nm^{-3})*	
	Legislative limit	Average 2021	Legislative limit	Average 2021
As	0.15	0.007	0.5	0.0003
Cd	0.001	0.0001	0.03** (0.05)	0.00004
Co	-†	-†	0.5	0.00007
Cr	0.05	0.005	0.5	0.003
Cu	0.5	0.001	0.5	0.001
Hg	0.001	0.0002	0.03** (0.05)	0.002
Mn	-†	-†	0.5	0.002
Ni	0.2	0.004	0.5	0.004
Pb	0.05	0.0008	0.5	0.0006
Sb	-	-†	0.5	0.0002
Tl	0.05	0.0009	0.05	0.00006
V	-†	-†	0.5	0.0002
Zn	0.5	0.006	-†	-†
pH	6.5-9.0	8.1	-†	-†
HCL				0.18
HF				0.006
NH3				0.02
PCDD/F	0.3 (ng I-TEQ·L^{-1})	0.003 (ng I-TEQ·L^{-1})	0.1 (ng I-TEQ·Nm^{-3})	0.005 (ng I-TEQ·Nm^{-3})

* Nm^3: flue gas normalized to dry gas and 11 % O_2, validated according to SFS 2013:253
**: Locally enforced limit value, value from SFS 2013: 253 in brackets, †: Not measured in this compartment
Values were obtained from the facility's annual environmental report to the Swedish EPA.

The internal water treatment consists of two main sections – the first one removes heavy metals and other contaminants from the acidic scrubber water, while the second one removes harmful substances from the flue gas condensate. The first section has a flow that varies between 1.5 to 20 $m^3 \cdot h^{-1}$, and consists of pH-adjustment, a CO_2-stripper, precipitation of heavy metals using TMT-15 (15 % water solution of $C_3N_3S_3Na_3$; CAS 17766-26-6, Algol Chemicals, Esbo, Finland), flocculation using a sulphur group functionalized organic polymer Kurifloc 6504 (Kurita Europe, Mannheim, Germany) and a lamella clarifier. After the lamella clarifier, water from the condensate scrubber is introduced to the water treatment and another set of precipitation, flocculation and lamella clarification is performed at a flow rate between 4 to 14 $m^3 \cdot h^{-1}$. The setup is constructed to allow the acidic scrubber condensate to be treated twice, even if a notable dilution occurs when the condensate is introduced into the process. The final stage of the water treatment process is a sand filter. Solid waste from the process water treatment (from the precipitation steps and lamella clarifiers) is mixed with boiler ash and filter ash to form self-hardening APCR. The APCR is landfilled at a hazardous waste landfill.

The addition of sludge during the Sludge:MSWI sampling campaign had no detrimental effect on the incineration, district heating production or electricity generation. The average incineration temperature during the Sludge:MSWI campaign was 910 - 1089 °C, and during the MSWI campaign it was 899 - 1082 °C, both well above the legislative limit.

Section S2. Flue gas sampling.

Flue gas sampling was conducted before the quench over a period of six hours per sampling occasion, maintaining a flow rate of approximately 16 L min^{-1}. In total, six sampling

occasions were conducted: three with the typical municipal solid waste mix, and three with the addition of 5-8wt% sludge from a wastewater treatment plant to the waste fuel.

The sampling method is based on method EN 1948:1 (designed for dioxin sampling) (Fig. S1). The sampling train consists of a cooled glass probe (8 mm i.d.) that is inserted in the centre of the flue gas duct. The flue gases are led to a impinge bottle containing 250 mL of MilliQ water. Prior to the sampling, the MilliQ water was spiked with 3 ng isotopically labelled standard to account for losses during sampling. The gases are then led to an impinge bottle containing 200 mL of 0.1 M sodium hydroxide. Both bottles are placed in an ice bath to facilitate the condensation of any water vapor present in the flue gas.

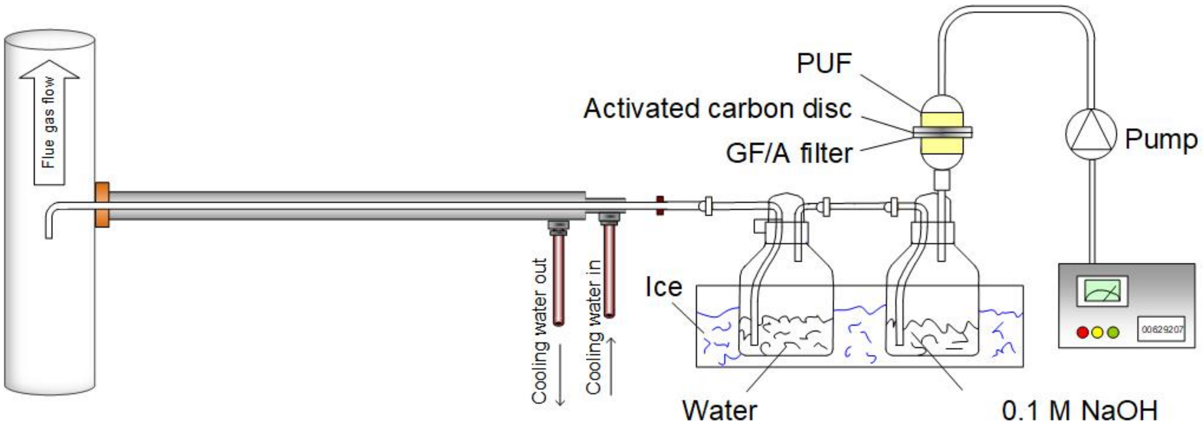

Figure S1. Flue gas sampling train.

Following the impinger bottles, there is a filter holder housing a pre-cleaned activated carbon disc (47 mm x 2 mm, Futamura Chemical CO. LTD. Nagoya. Aichi. Japan) and a GF/A filter (47 mm diameter, pore size 1.6 µm, VWR International, Radnor, PA, USA), positioned between two polyurethane foam filters (47 mm diameter, height 50 mm, Sunde Skumplast AS, Gan, Norway).

To facilitate comparison of concentrations in the flue gas, the volume of sampled flue gas was normalized to dry gas, 0°C and 1 atm pressure according to Equation S1.

Equation S1.

$$Normalized\ gas\ volume = V_{pump} \times p_{air} \times \frac{t_{0°C}}{t_{pump} \times t_{0°C}} \times (1 - \frac{\%H_2O}{100})$$

V_{pump} = Gas volume sampled (m^3); p_{air} = Air pressure at sampling (atm)

t_{pump} = Temperature at sampling (°C); $t_{0°C}$ = Temperature at 0°C (K)

$\%H_2O$ = % of H_2O in sampled flue gas

Section S3. Sampling protocol.

Sampling was performed on a total of six days. During three of these days, the standard mix of municipal solid waste was incinerated, and during three days 5-8% of sewage sludge was added to the waste mixture.

Table S2. Sample collection protocol.

Sample name	Sample collection	Sampling method	# of samples collected MSWI	# of samples collected Sludge:MSWI
Untreated flue gas	6 h/ day	Modified method EN 1948:1	3	3
Condensate	3 subsamples/ day, pooled	HDPE bottles, pre-rinsed with MQ	3	3
Boiler ash	3 subsamples/ day, pooled	Stainless steel containers	3	3
Filter ash	3 subsamples/ day, pooled	Stainless steel containers	3	3

The solid samples were mixed and quartered in the lab to make sure that subsamples were representative. The bottom ash was very heterogenous, and had to be ball milled after quartering to achieve better homogeneity.

Section S4. Instrumental parameters, quality assurance

Table S3. Target compounds included in the analysis, their chemical formula, parent ion, quantification ion and the corresponding internal and recovery standard.

Acronym	Compound	Formula	Parent ion (m/z)	Quantification ion (m/z)	Qualification ion (m/z)	Internal standard
**PFCAs**	**Perfluorocarboxylic acids**					
PFBA	Perfluorobutanoic acid	$C_3F_7CO_2H$	212.98	168.99	-	M4PFBA
PFPeA	Perfluoropentanoic acid	$C_4F_9CO_2H$	262.98	218.99	-	M2PFHxA
PFHxA	Perfluorohexanoic acid	$C_5F_{11}CO_2H$	312.97	268.99	118.95	M2PFHxA
PFHpA	Perfluoroheptanoic acid	$C_6F_{13}CO_2H$	362.97	318.98	167.97	M2PFOA
PFOA	Perfluorooctanoic acid	$C_7F_{15}CO_2H$	412.97	368.98	168.97	M2PFOA
PFDA	Perfluorodecanoic acid	$C_9F_{19}CO_2H$	512.97	468.97	219.00	M2PFDA
PFDoDA	Perfluorododecanoic acid	$C_{11}F_{23}CO_2H$	612.97	568.96	168.96	M2PFDA
PFTeDA	Perfluorotetradecanoic acid	$C_{13}F_{27}CO_2H$	712.95	668.96	168.97	M2PFDA
**PFSAs**	**Perfluorosulphonic acids**					
PFBS	Perfluorobutanesulphonic acid	$C_4F_9SO_3H$	298.95	79.96	79.96	M3PFHxS
PFPeS	Perfluoropentanesulphonic acid	$C_5F_{11}SO_3H$	348.94	79.96	80.00	M3PFHxS
PFHxS	Perfluorohexanesulphonic acid	$C_6F_{13}SO_3H$	398.94	79.96	98.90	M3PFHxS
PFHpS	Perfluoroheptanesulphonic acid	$C_7F_{15}SO_3H$	448.94	98.96	79.96	M4PFOS
PFOS	Perfluorooctanesulphonic acid	$C_8F_{17}SO_3H$	498.93	98.96	79.96	M4PFOS
PFDoDS	Perfluorododecanesulphonic acid	$C_{12}F_{25}SO_3H$	698.92	98.96	79.96	M4PFOS
**FTSAs**	**Fluorotelomer sulphonic acids**					
6:2 FTSA	6:2 fluorotelomersulphonic acid	$C_8F_{13}H_4SO_3H$	426.97	80.96	81.00	M2-6:2FTSA
8:2 FTSA	8:2 fluorotelomersulphonic acid	$C_{10}F_{17}H_4SO_3H$	526.96	80.96	81.00	M2-6:2FTSA
**diPAPs**	**Polyfluoroalkyl phosphoric acid diesters**					
6:2diPAP	6:2 Fluorotelomer phosphate diester	$C_{16}H_9F_{26}O_4P$	788.98	442.97	96.97	M4PFOS
8:2diPAP	8:2 Fluorotelomer phosphate diester	$C_{20}H_9F_{34}O_4P$	988.97	542.97	96.97	M4PFOS

Table S3, continued

Acronym	Compound	Parent ion (m/z)	Quantification ion (m/z)	Recovery standard
**Internal standards**				
M4PFBA	Perfluoro-n-[1,2,3,4-$^{13}C_4$] butanoic acid	215.97	171.99	M8PFOA
M2PFHxA	Perfluoro-n-[1,2-$^{13}C_2$] hexanoic acid	314.97	269.99	M4PFOA
M2PFOA	Perfluoro-n-[1,2-$^{13}C_4$] octanoic acid	414.97	371.98	M8PFOA
M2PFDA	Perfluoro-n-[1,2-$^{13}C_2$] decanoic acid	514.97	469.97	M8PFOA
M3PFHxS	Perfluoro-1-[(1,2,3-$^{13}C_3$] hexanesulphonic acid	401.95	98.96	M8PFOS
M4PFOS	Perfluoro-1-[1,2,3,4-$^{13}C_4$]octanesulphonic acid	502.95	98.96	M8PFOS
M2-6:2FTSA	1H,1H,2H,2H-perfluoro-1-[1,2-$^{13}C_2$]-octane sulphonic acid	428.98	80.96	M8PFOS
**Recovery standards**				
M4PFOA	Perfluoro-n-[1,2,3,4-$^{13}C_4$] octanoic acid	416.98	171.99	
M8PFOS	Perfluoro-1-[$^{13}C_8$]octanesulphonic acid	506.96	98.96	

Table S4. Limit of detection per sample matrix.

	Filter ash	Boiler ash	Condensate	Untreated flue gas
	$ng{\cdot}g^{-1}$	$ng{\cdot}g^{-1}$	$ng{\cdot}L^{-1}$	$ng{\cdot}m^{-3}$
**PFBA**	0.25	0.25	1.2	0.07
**PFPeA**	0.02	0.02	0.11	0.01
**PFHxA**	0.05	0.05	0.24	0.01
**PFHpA**	0.04	0.04	0.19	0.01
**PFOA**	0.17	0.16	0.79	0.05
**PFDA**	0.06	0.06	0.29	0.02
**PFDoDA**	0.61	0.59	2.9	0.17
**PFTeDA**	0.42	0.41	2.0	0.12
**PFBS**	0.07	0.06	0.31	0.02
**PFPeS**	0.01	0.01	0.04	0.02
**PFHxS**	0.01	0.01	0.07	0.03
**PFHpS**	0.01	0.01	0.05	0.03
**PFOS**	0.02	0.02	0.08	0.02
**PFDoDS**	0.64	0.62	3.0	0.18
**6:2FTS**	0.32	0.32	1.5	0.09
**8:2FTS**	0.20	0.19	0.94	0.06
**6:2diPAP**	1.1	1.0	5.1	0.30
**8:2diPAP**	1.3	1.3	6.3	0.37

Table S5. Concentrations of PFAS in all samples.

	Unit	PFBA	PFPeA*	PFHxA	PFHpA*	PFOA	PFDA	PFBS*	PFOS	Sum
Untreated flue gas MSWI day 1		3.7	<LOD	2.7	*0.06*	0.71	<LOD	*0.07*	<LOD	7.3
Untreated flue gas MSWI day 2	$ng{\cdot}m^{-3}$	2.4	<LOD	2.4	0.09	0.22	<LOD	*0.03*	<LOD	5.2
Untreated flue gas MSWI day 3		2.8	<LOD	6.1	0.15	0.39	<LOD	*0.04*	<LOD	9.5
Average		3.0	<LOD	3.7	0.10	0.44	<LOD	*0.04*	<LOD	7.3
Untreated flue gas Sludge:MSWI day 1		2.7	0.11	7.0	0.49	0.66	0.12	0.10	<LOD	11
Untreated flue gas Sludge:MSWI day 2	$ng{\cdot}m^{-3}$	3.5	0.13	13	0.95	1.3	0.37	*0.03*	<LOD	19
Untreated flue gas Sludge:MSWI day 3		2.4	0.15	8.1	0.55	0.73	0.20	*0.06*	<LOD	12
Average		2.9	0.13	9.3	0.66	0.88	0.23	0.06	<LOD	14
Condensate MSWI day 1		<LOD	<LOD	46	1.9	1.9	<LOD	<LOD	<LOD	50
Condensate MSWI day 2	$ng{\cdot}L^{-1}$	<LOD	<LOD	42	1.8	2.0	<LOD	<LOD	<LOD	46
Condensate MSWI day 3		<LOD	<LOD	46	1.7	1.5	<LOD	<LOD	<LOD	49
Average		<LOD	<LOD	45	1.8	1.8	<LOD	<LOD	<LOD	48
Condensate Sludge:MSWI day 1		<LOD	<LOD	90	3.3	3.7	*0.76*	<LOD	<LOD	98
Condensate Sludge:MSWI day 2	$ng{\cdot}L^{-1}$	<LOD	<LOD	85	4.1	5.1	1.4	<LOD	<LOD	95
Condensate Sludge:MSWI day 3		<LOD	<LOD	109	5.4	4.4	*0.35*	<LOD	<LOD	119
Average		<LOD	<LOD	95	4.3	4.4	0.83	<LOD	<LOD	104
Filter ash MSWI day 1		<LOD	<LOD	0.28	<LOD	<LOD	<LOD	<LOD	<LOD	0.28
Filter ash MSWI day 2	$ng{\cdot}g^{-1}$	<LOD	<LOD	0.60	<LOD	*0.18*	<LOD	<LOD	<LOD	0.79
Filter ash MSWI day 3		<LOD	<LOD	0.70	<LOD	<LOD	<LOD	<LOD	<LOD	0.70
Average		<LOD	<LOD	0.53	<LOD	*0.06*	<LOD	<LOD	<LOD	0.59
Filter ash Sludge:MSWI day 1		<LOD	<LOD	0.40	<LOD	0.38	<LOD	<LOD	<LOD	0.77
Filter ash Sludge:MSWI day 2	$ng{\cdot}g^{-1}$	<LOD	<LOD	*0.13*	<LOD	<LOD	<LOD	<LOD	<LOD	0.13
Filter ash Sludge:MSWI day 3		<LOD	<LOD	0.37	<LOD	<LOD	<LOD	<LOD	<LOD	0.37
Average		<LOD	<LOD	0.30	<LOD	0.13	<LOD	<LOD	<LOD	0.43
Boiler ash MSWI day 1		<LOD	<LOD	<LOD	<LOD	<LOD	<LOD	<LOD	<LOD	<LOD
Boiler ash MSWI day 2	$ng{\cdot}g^{-1}$	<LOD	<LOD	<LOD	<LOD	<LOD	<LOD	<LOD	<LOD	<LOD
Boiler ash MSWI day 3		<LOD	<LOD	<LOD	<LOD	<LOD	<LOD	<LOD	<LOD	<LOD
Average		<LOD	<LOD	<LOD	<LOD	<LOD	<LOD	<LOD	<LOD	<LOD
Boiler ash Sludge:MSWI day 1		*0.67*	<LOD	<LOD	<LOD	<LOD	<LOD	<LOD	<LOD	0.67
Boiler ash Sludge:MSWI day 2	$ng{\cdot}g^{-1}$	*0.40*	<LOD	<LOD	<LOD	<LOD	<LOD	<LOD	<LOD	0.48
Boiler ash Sludge:MSWI day 3		<LOD	<LOD	<LOD	<LOD	<LOD	<LOD	1.16	<LOD	1.16
Average		*0.36*	<LOD	<LOD	<LOD	<LOD	<LOD	0.39	<LOD	0.75

Concentrations below the method detection limit is marked by <LOD. Numbers in italics are above LOD but below limit of quantification (LOQ). Results <LOD were treated as zero when calculating sums and averages. *Compound was quantified using the internal standard closest in retention time and levels should be considered semi-quantitative.

Table S6. Concentration of PFAS in field blanks.

	Unit	PFBA	PFPeA	PFHxA	PFHpA	PFOA	PFDA	PFDoDA	PFTeDA	PFBS
**Condensate MSWI**	$ng\cdot L^{-1}$	<LOD	<LOD	<LOD	<LOD	<LOD	<LOD	<LOD	<LOD	<LOD
**Condensate Sludge:MSWI**		<LOD	<LOD	<LOD	<LOD	<LOD	<LOD	<LOD	<LOD	<LOD
**Untreated flue gas MSWI**	$ng\cdot m^{-3}$	1.2	<LOD	0.75	0.11	0.37	<LOD	<LOD	<LOD	0.12
**Untreated flue gas Sludge:MSWI**		1.1	<LOD	<LOD	<LOD	*0.26*	0.09	<LOD	<LOD	0.20

	Unit	PFPeS	PFHxS	PFHpS	PFOS	PFDoDS	6:2FTS	8:2FTS	6:2diPAP	8:2diPAP
**Condensate MSWI**	$ng\cdot L^{-1}$	<LOD	<LOD	<LOD	<LOD	<LOD	<LOD	<LOD	<LOD	<LOD
**Condensate Sludge:MSWI**		<LOD	<LOD	<LOD	<LOD	<LOD	<LOD	<LOD	<LOD	<LOD
**Untreated flue gas MSWI**	$ng\cdot m^{-3}$	<LOD	<LOD	<LOD	<LOD	<LOD	<LOD	<LOD	<LOD	<LOD
**Untreated flue gas Sludge:MSWI**		<LOD	<LOD	<LOD	<LOD	<LOD	<LOD	<LOD	<LOD	<LOD

Table S7. Average internal standard recoveries per sample matrix.

	PFBA	PFHxA	PFOA	PFDA	PFHxS	PFOS	6:2FTS
Blank	92% ±18%	83% ±11%	85% ±7%	89% ±18%	116% ±27%	108% ±23%	149% ±39%
Filter ash	81% ±8%	78% ±5%	86% ±5%	78% ±10%	122% ±20%	115% ±26%	96% ±20%
Condensate	101% ±7%	100% ±8%	90% ±2%	87% ±7%	140% ±10%	133% ±7%	168% ±13%
Boiler ash	77% ±7%	71% ±7%	81% ±5%	77% ±%12%	111% ±26%	105% ±25%	73% ±14%
MilliQ Flue gas	72% ±28%	105% ±29%	81% ±13%	73% ±17%	88% ±10%	97% ±25%	90% ±24%
NaOH Flue gas	70% ±28%	89% ±25%	75% ±15%	66% ±26%	79% ±26%	80% ±45%	85% ±11%
Filter Flue gas	45% ±17%	42% ±13%	43% ±13%	44% ±16%	58% ±27%	61% ±25%	128% ±66%
**Average**	76% ±16%	81% ±19%	75% ±14%	69% ±13%	95% ±28%	97% ±22%	102% ±32%

Table S8. Recovery of flue gas sampling spikes.

	Recovery Sampling Spike (M8PFOS)
Untreated flue gas MSWI day 1	53%
Untreated flue gas MSWI day 2	37%
Untreated flue gas MSWI day 3	46%
Untreated flue gas Sludge:MSWI day 1	105%
Untreated flue gas Sludge:MSWI day 2	86%
Untreated flue gas Sludge:MSWI day 3	64%

Table S9. Relative standard deviation of replicates per sample.

	PFBA	PFHxA	PFHpA	PFOA	PFDA
**Filter ash MSWI (n = 3)**	<LOD	18%	<LOD	<LOD	<LOD
**Filter ash Sludge:MSWI (n = 3)**	<LOD	4%	<LOD	<LOD	<LOD
**Boiler ash MSWI (n = 3)**	<LOD	<LOD	<LOD	<LOD	<LOD
**Boiler ash Sludge:MSWI (n = 3)**	n/a[1]	<LOD	<LOD	n/a[2]	<LOD
**Condensate MSWI (n = 2)**	<LOD	1%	1%	5%	<LOD
**Condensate Sludge:MSWI (n = 2)**	<LOD	5%	3%	5%	n/a[2]

[1]Detected in 2 replicates, but below LOQ. [2]Detected in 1 replicate, but below LOQ.

Tabell S10. Concentration of PFAS in procedural blanks (ng·L^{-1}).

Compound	PFBA	PFPeA
**Blank 1**	1.6	<LOD
**Blank 2**	7.4	<LOD
**Blank 3**	<LOD	<LOD
**Blank 4**	<LOD	<LOD
**Blank 5**	<LOD	<LOD
**Blank 6**	<LOD	<LOD
**Blank 7**	<LOD	<LOD
**Blank 8**	<LOD	<LOD
**Blank 9**	<LOD	<LOD
**Blank 10**	<LOD	<LOD
**Blank 11**	<LOD	<LOD
**Blank 12**	<LOD	<LOD
**Blank 13**	<LOD	<LOD
**Blank 14**	<LOD	<LOD
**Blank 15**	<LOD	<LOD
**Blank 16**	<LOD	<LOD
**Blank 17**	<LOD	<LOD
**Blank 18**	<LOD	<LOD

PFDoDS	PFOS	PFHpS	PFHxS	PFPeS	PFBS	PFTeDA	PFDoDA	PFDA	PFOA	PFHpA	PFHxA
<LOD	<LOD	<LOD	<LOD	<LOD	<LOD	<LOD	<LOD	<LOD	<LOD	<LOD	<LOD
<LOD	<LOD	<LOD	<LOD	<LOD	<LOD	<LOD	<LOD	<LOD	<LOD	<LOD	<LOD
<LOD	<LOD	<LOD	<LOD	<LOD	<LOD	<LOD	<LOD	<LOD	<LOD	<LOD	<LOD
<LOD	<LOD	<LOD	<LOD	<LOD	<LOD	<LOD	<LOD	<LOD	<LOD	<LOD	<LOD
<LOD	<LOD	<LOD	<LOD	<LOD	<LOD	<LOD	<LOD	<LOD	<LOD	<LOD	<LOD
<LOD	<LOD	<LOD	<LOD	<LOD	<LOD	<LOD	<LOD	<LOD	<LOD	<LOD	<LOD
<LOD	<LOD	<LOD	<LOD	<LOD	<LOD	<LOD	<LOD	<LOD	<LOD	<LOD	<LOD
<LOD	<LOD	<LOD	<LOD	<LOD	<LOD	<LOD	<LOD	<LOD	<LOD	<LOD	<LOD
<LOD	<LOD	<LOD	<LOD	<LOD	<LOD	<LOD	<LOD	<LOD	<LOD	<LOD	<LOD
<LOD	<LOD	<LOD	<LOD	<LOD	<LOD	<LOD	<LOD	<LOD	<LOD	<LOD	<LOD
<LOD	<LOD	<LOD	<LOD	<LOD	<LOD	<LOD	<LOD	<LOD	<LOD	<LOD	<LOD
<LOD	<LOD	<LOD	<LOD	<LOD	<LOD	<LOD	<LOD	<LOD	<LOD	<LOD	<LOD
<LOD	<LOD	<LOD	<LOD	<LOD	<LOD	<LOD	<LOD	<LOD	<LOD	<LOD	<LOD
<LOD	<LOD	<LOD	<LOD	<LOD	<LOD	<LOD	<LOD	<LOD	<LOD	<LOD	<LOD
<LOD	<LOD	<LOD	<LOD	<LOD	<LOD	<LOD	<LOD	<LOD	<LOD	<LOD	<LOD
<LOD	<LOD	<LOD	<LOD	<LOD	<LOD	<LOD	<LOD	<LOD	<LOD	<LOD	<LOD
<LOD	<LOD	<LOD	<LOD	<LOD	<LOD	<LOD	<LOD	<LOD	<LOD	<LOD	<LOD
<LOD	<LOD	<LOD	<LOD	<LOD	<LOD	<LOD	<LOD	<LOD	<LOD	<LOD	<LOD

Compound	8:2diPAP	6:2diPAP	8:2FTS	6:2FTS
**Blank 1**	<LOD	<LOD	<LOD	<LOD
**Blank 2**	<LOD	<LOD	<LOD	<LOD
**Blank 3**	<LOD	<LOD	<LOD	<LOD
**Blank 4**	<LOD	<LOD	<LOD	<LOD
**Blank 5**	<LOD	<LOD	<LOD	<LOD
**Blank 6**	<LOD	<LOD	<LOD	<LOD
**Blank 7**	<LOD	<LOD	<LOD	<LOD
**Blank 8**	<LOD	<LOD	<LOD	<LOD
**Blank 9**	<LOD	<LOD	<LOD	<LOD
**Blank 10**	<LOD	<LOD	<LOD	<LOD
**Blank 11**	<LOD	<LOD	<LOD	<LOD
**Blank 12**	<LOD	<LOD	<LOD	<LOD
**Blank 13**	<LOD	<LOD	<LOD	<LOD
**Blank 14**	<LOD	<LOD	<LOD	<LOD
**Blank 15**	<LOD	<LOD	<LOD	<LOD
**Blank 16**	<LOD	<LOD	<LOD	<LOD
**Blank 17**	<LOD	<LOD	<LOD	<LOD
**Blank 18**	<LOD	<LOD	<LOD	<LOD

Table S11. Flue gas sampling parameters.

Parameter	MSWI (min-max)	Sludge:MSWI (min-max)
Air pressure (atm)	0.99–1.0	0.98–0.99
Pump temperature (°C)	25°C	25°C
% H_2O wet gas	15-17	14-18
% O_2 wet gas	5.8-11	5.6-8.2

Table S12. LC-MS instrument parameters.

Parameter	Value
**Gas Temp (°C)**	250
**Gas Flow (L/min)**	5
**Nebulizer (psig)**	30
**SheathGasTemp (°C)**	350
**SheathGasFlow (L/min)**	12
**Injection volume (µL)**	10.0

Table S13. LC mobile phase program.

	Time	A (2 mM NH_4Ac in H_2O)	B (2 mM NH_4Ac in MeOH)	Flow
**1**	2.00 min	70.00 %	30.00 %	0.5 mL/min
**2**	14.00 min	0.00 %	100.00 %	0.5 mL/min
**3**	17.00 min	0.00 %	100.00 %	0.5 mL/min

References

(1) Björklund, S.; Weidemann, E.; Jansson, S. Emission of Per- and Polyfluoroalkyl Substances from a Waste-to-Energy Plant - Occurrence in Ashes, Treated Process Water, and First Observation in Flue Gas. *Environmental science & technology* **2023**, *57* (27), 10089-10095. DOI: 10.1021/acs.est.2c08960.
